# How does language change as a lexical network? An investigation based on written Chinese word co-occurrence networks

**DOI:** 10.1371/journal.pone.0192545

**Published:** 2018-02-28

**Authors:** Heng Chen, Xinying Chen, Haitao Liu

**Affiliations:** 1 Center for Linguistics and Applied Linguistics, Guangdong University of Foreign Studies, Guangzhou, China; 2 School of Foreign Studies, Xi’an Jiaotong University, Xi’an, China; 3 Department of Linguistics, Zhejiang University, Hangzhou, China; 4 Ningbo Institute of Technology, Zhejiang University, Ningbo, China; Universidad Rey Juan Carlos, SPAIN

## Abstract

Language is a complex adaptive system, but how does it change? For investigating this process, four diachronic Chinese word co-occurrence networks have been built based on texts that were written during the last 2,000 years. By comparing the network indicators that are associated with the hierarchical features in language networks, we learn that the hierarchy of Chinese lexical networks has indeed evolved over time at three different levels. The connections of words at the micro level are continually weakening; the number of words in the meso-level communities has increased significantly; and the network is expanding at the macro level. This means that more and more words tend to be connected to medium-central words and form different communities. Meanwhile, fewer high-central words link these communities into a highly efficient small-world network. Understanding this process may be crucial for understanding the increasing structural complexity of the language system.

## Introduction

Language is the main evolutionary contribution of humans [1, 2], and is intimately interwoven with cultural evolution and our superior mental functions [3]. Many studies show that language is a complex adaptive system (CAS) [4, 5, 6, 7], possessing features such as hierarchical structures in syntactic organizations [8, 9], scale-free and small-world properties in language networks [10], and the well-known “principle of least effort” of Zipf’s law [11]. As complex systems, “all languages are complex to some degree” [12, 13], and “the older a language, the more complexity it has” [14]. The question we would like to address here is how a language as a complex system, to be specific, lexical network, changes over time.

Hierarchy is considered to be an important feature of complex systems [15], and the understanding of the relationship between hierarchies has been a key concern in complex system research. In the latest approach—namely, complex network analysis—hierarchies are even considered one of the key elements that lead to the complexity of network systems, such as small-world and scale-free features [16]. Simon [17] proclaims that 1) the complex system must be hierarchical; 2) the origin of the hierarchical structure is the interaction between agents; and 3) adaptation and hierarchy are implicitly coupled features.

Modern linguists also believe that the hierarchical nature of language is one of the essential attributes of languages [8, 18]. Existing studies discussing the language hierarchies are multifarious and are mainly concerned with problems that occur in at a certain hierarchical level, such as understanding the patterns of combining words to phrases. In contrast, the CAS theory recognizes the relativity and complexity of language hierarchies, thereby bringing fresh insights to the question. It can address the macro and micro features simultaneously, and balance the discussions on both sides as well [19, 20]. Moreover, the CAS theory treats the states of the system as parts or results of an evolving process, which provides a new way for us to understand the adaptability and complexity of the language system. Therefore, the evolution of the language hierarchy feature can be seen, also, as the process of language adaptation. However, it needs to be noticed that this hierarchy feature is not equivalent to the syntactic hierarchy feature in Linguistics. The hierarchy feature we explored here is from the perspective of local connectivity, modular community structure, and the global connectivity of a network, e.g., as applied in [21, 22, 23]. Moreover, the classical hierarchy feature of Degree Dependent Clustering Coefficient in language [24] is also observed.

Complex network analysis is a very effective tool for studying complex systems [19], and it has been increasingly applied in linguistic studies (for a review, see [10, 25]). A complex network is a model of a system [26] and dynamic network constitutes a large part of CAS theory [27]. In the current study, complex network analysis offers a feasible way of discussing the hierarchy question in languages from a more systematic way, which describes the macroscopic and microscopic levels of the language system simultaneously [20].

Applying complex network models to study the dynamic features of language systems has become popular in recent years in fields such as language evolution/change and language development [2, 28, 29], quantitative linguistics [30, 31, 32, 33, 34], and language acquisition [21, 35, 36]. With regard to word co-occurrence networks, there have been many studies [29, 30, 35, 37–39]. Among them, Ferrer-i-Cancho, Solé and Köhler [24] verified the existence of hierarchical features in the English syntactic network and Liu and Cong [40] found that hierarchical features also exist in the Chinese syntactic and semantic networks; in addition, they also found out that there are differences between the subsystems of these two kinds of networks.

However, research on the change of language hierarchy has been still absent until now. To explore how language evolves, we need to investigate a manageable number of reliable diachronic texts. Although there is usually a gap between written and spoken language, especially in Sinitic languages, most of our findings about languages, even in modern times, depend much on written records [41, 42]. In this study, we reason that written Chinese is suitable for our exploration [43, 44, 45], since it is one of the most archaic living languages for which we have a more or less continuous written record. This study constructs and analyzes four diachronic Chinese word co-occurrence networks. It aims to discover the macroscopic change patterns appearing in the Chinese language system by observing and comparing the hierarchical characteristics of four diachronic networks. Furthermore, we will combine our results with common quantitative linguistic measurements such as utterance length distribution and word length distribution and attempt to reveal and explain the causes and internal mechanism of the origin and the change of language hierarchies.

The questions that will be discussed in the current study include:

1. Has the Chinese word co-occurence network always been a complex network? If so, then how does it change as a complex network?
2. How has the Chinese word co-occurence network changed from the perspective of network hierarchy? And what is the linguistic significance of this change?
3. How does the length distribution of clauses and words change in written Chinese? What is the significance of this change with regard to the network change?
4. What is the most likely motivation and mechanism of the change of the Chinese word co-occurence network?

The study is organized as follows: First, the construction method of the word co-occurrence network and the acquisition and processing of the language network parameters are introduced in Section 2. Then, the parameters of the four diachronic language networks and some related language units are compared in Section 3. Section 4 is devoted to a discussion of the results. The patterns of change and the mechanism behind it will be explored in depth in this section. The study is concluded in Section 5.

## Data and methods

### Data selection and preparation

For ensuring that this study reflects the real dynamic change of the Chinese language, we have chosen authentic texts that are relatively closer to the spoken language during different times in history (for details on the selected texts, see Table 1). It is critical to choose appropriate ancient texts since there is an evident distinction between two kinds of Chinese written texts: the mandarin Chinese and the classic Chinese [46]. The former is much closer to spoken language, and the latter is an independent system that did not change much since pre-Qin dynasty (about from B.C. 3 to Pre-A.D. 20). This study includes short narrative texts of mandarin Chinese from four historical periods, which are representative for language used in their times [42, 45, 47]. Specifically, the narrative texts from Time Period 1 to 3 are from http://www.gushiwen.org/, and the Xinwen Lianbo texts in Time Period 4 are from http://tv.cctv.com/lm/xwlb/. To ensure the comparability, the text length has been fixed at 10,000 tokens, which means the four networks may have different sizes. Even so, we attributed the parameter differences between four networks to the change of Written Chinese per se, to be specific, the birth, use and death of words in language change [48].

**Table 1:** Selected texts in the corpus.

Corpus information	Corpus 1	Corpus 2	Corpus 3	Corpus 4
	Ancient	Middle Ancient	Modern Times	Modern
Name of the text	孟子 *mengzi*	颜氏家训书 *yanshi jiaxun shu*	宋元白话小说 *song yuan baihua xiaoshuo*	新闻联播 *xinwen lianbo*
Size (words)	10,000	10,000	10,000	10,000
Total of word types	1,162	1,945	1,421	1,370
Total of characters	1,165	12,640	13,457	17,854
Time Period	B.C. 5–B.C. 3	A.D. 4–A.D. 6	A.D. 12–A.D. 14	A.D. 21

Genre is also an essential factor of texts. We only select short narrative stories from the same register for this study. Since there is no ready-made word segmentation tool for ancient Chinese, we commissioned experts on ancient Chinese to manually segment the texts. The chief problem of manual segmentation is selecting the criteria for wordhood, especially when it comes to the distinction between words and phrases [49]. Through manual proofreading and correction of the segmentation results, we ensure that the accuracy rate of word segmentation is at least 98%. The total amount of Chinese characters in each of these texts is different due to the change of word length over time. However, words are the actual basic units of language usage, not characters. Thus, it does not matter whether they are mono syllable/character words in ancient Chinese (e.g., 道, *dao*, means road, morality, doctrine, or method) or two syllable/character words in modern Chinese (e.g., 电话, *dianhua*, means telephone); both of them are the basic units of language used in their times.

### Word co-occurrence networks

We construct the networks based on the linear relation of words in the authentic texts. It is worth noting that before building the networks, we must deal with the punctuation problem. Punctuation such as “,”, “!”, “.”, “…”, “:”, and “;” are treated as signals to stop, while other punctuation such as “《》”, “**“”**”, “·”, “『』”, and “【】”, are dealt with according on the context. We refer to the words sequences that are separated by punctuation as “linguistic blocks” in this study. The loops, the arcs between two tokens that share the same word type, are allowed in the networks.

Why do we choose the “word co-occurrence” networks? Miyagawa, Berwick and Okanoya argued that the language system is composed of two modules—namely, L(exical) and E(xpression)—and expressions are the combinations of vocabulary/words [9]. Meara defined the co-occurrence relationship of words as the “co-citation links of words in a text” [50]. Furthermore, she claimed that word clustering, which is caused by word co-occurrence relationships, is perhaps the embodiment of this emerging feature. In other words, the co-occurrence relationships of words reflect the nature of a text, and they can generate some very interesting and rather unusual mappings of text structures. Furthermore, word co-occurence models have also been applied to authorship attribution [51], semantic analyses [52] and word clustering [53]. It is a widely used text feature from the linguistic point.

**Fig 1:**
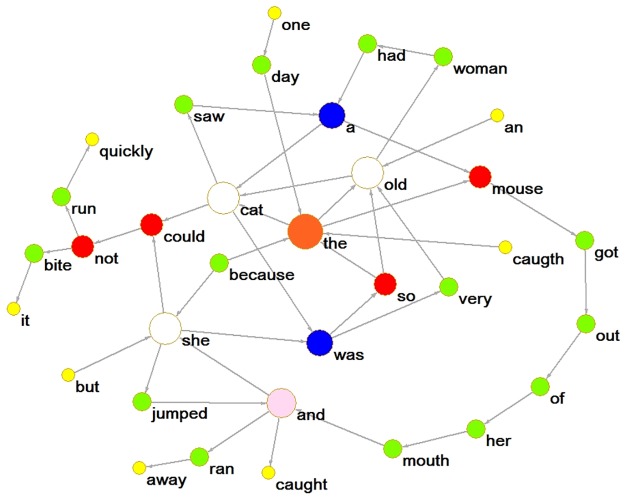
A word-form co-occurrence network based on an English paragraph.

We run java codes, 2-gramWordCount, for obtaining the adjacent word pairs or 2-grams. For illustration, an English network sample based on a simple English paragraph (S1 File) is given in Fig 1. The nodes in Fig 1 are word forms. However, since there is no distinction between word forms and word types in Chinese, there is only one word type for a Chinese text.

We used Cytoscape and Pajek to obtain the basic parameters. There are 34 nodes and 44 arcs in the network. The more in-depth parameters are displayed below.

### Network parameters

The network parameters that we are most interested in are those closely related to language hierarchical features, such as clustering coefficient (<*C>*), degree-dependent clustering coefficient $\bar{C}\left(k\right)$, average path length (*<l>*), degree (*Q*), and modularity. Some common network parameters, such as, the power rate index of the degree distribution, average degree of nearest neighbors ${\bar{K}}_{n}{}_{n}\left(k\right)$, diameter (*D*), density, centrality, etc. are also investigated for comparison. For better understanding of the results, the non-network parameters, mean utterance length (MUL) and average word length are also applied in current study. All the network parameters are calculated by network analysis software Cytoscape and Pajek.

#### Degree distribution and average degree of nearest neighbors

The degree *k* of a node is the number of its neighbors. For example, the degree of the node “she” in Fig 1 is 6, and the degree of the node “woman” is 2. If we distinguish the directions of the arcs between nodes, we can then obtain in-degrees and out-degrees. In-degree is the number of arcs that a node receives, and out-degree is the number of arcs that a node sends out. For example, the in-degree of the node “she” is 3, and its out-degree is 3 also; meanwhile, the node “woman” has an in-degree of 1, and an out-degree of 1 as well. The average degree of a network is the ratio of the sum of degrees of all nodes to the number of nodes.

The scale-free feature is one of the key characteristics of complex networks, that is, the probability distribution of nodes’ degrees *P(k)* fix to the power-law. More specifically, if nodes’ degrees are on the X-axis, and the numbers of nodes that share the same degrees on the Y-axis, then the data points scattering in the coordinate axis fix to the power law formula

$$P\left(k\right)\sim k^{-\alpha }$$

and the distribution *P(k)* has a linearly decreasing tendency. These kinds of networks are called scale-free networks. The scale-free feature actually means that there are only a few nodes with high degrees in the network, while the most nodes’ degrees are very low. The high-degree nodes are often the hubs in the network. Just as other networks in the real world, language complex networks are scale-free networks. Therefore, it means that only a few linguistic units in the language system have a strong ability to combine with other linguistic units, and most of the language units are relatively weak with this ability.

For investigating the influence of one node’s degree to its neighbors’ degrees, the parameter Average Degree of Nearest Neighbors ${\bar{K}}_{n}{}_{n}\left(k\right)$ can be applied. The ${\bar{K}}_{n}{}_{n}\left(k\right)$ is the average degrees of nodes whom are neighbors of nodes with degree *k*. It represents the correlation between the degree of a node and the degrees of its neighbors. If ${\bar{K}}_{n}{}_{n}\left(k\right)$ is an increasing function of *k* (or *r*> 0), then there is positive correlation/assortative mixing pattern in the network, which indicates that nodes with high degrees (low degrees) tend to be neighbors of high-degree (low-degree) nodes. On the contrary, if ${\bar{K}}_{n}{}_{n}\left(k\right)$ is a decreasing function of *k* (or *r* <0), then the network exhibits a negative correlation/disassortative mixing pattern indicating that nodes with high degrees (low degrees) tend to be neighbors of low-degree (high-degree) nodes. In addition, the ${\bar{K}}_{n}{}_{n}\left(k\right)$ distributions of the real world networks are usually fit to the power law ${\bar{K}}_{n}{}_{n}\left(k\right)\sim k^{-\gamma }$.

#### Small-world networks

Another crucial aspect of complex networks is the small-world feature. What is the small-world feature? According to Watts & Strogatz, it is defined by two complex network parameters, the clustering coefficient (*<C>*) and the average path length (*<l>*) [54]. The clustering coefficient is an index used to measure the tendency of aggregation or the tendency of forming clusters. It measures the possibility that a node’s neighbors are also connected to each other. Specifically, if *k*_*i*_ is the degree of node *i* (i.e., node *i* has *k*_*i*_ neighbors), and *E*_*i*_ is regarded as the edges between these *k*_*i*_ nodes, then these *k*_*i*_ nodes can maximally have *k*_*i*_(*k*_*i*_ − 1) / 2 edges and the clustering coefficient *C*_*i*_ of the node *i* is 2*E*_*i*_ / *k*_*i*_(*k*_*i*_ − 1). The clustering coefficient <*C*> of the whole network is therefore the mathematical mean of the clustering coefficients of all nodes. For example, as mentioned, the degree of the node “she” in Fig 1 is 6. Its six neighbors are *was*, *because*, *could*, *but*, *jumped*, and *and*. There is an edge between these six neighbors, *jumped-and*. Therefore, *E*_*i*_ is 1, and the maximum number of edges that can exist between these six neighbors is 15, so the clustering coefficient *C*_*i*_ of node “she” is approximately equal to 0.0667. It can be seen that the smaller the clustering coefficient, the sparser the network.

The average path length *<l>* measures the mean of all paths that connect any two nodes in the network. For a given pair of nodes *i*, *j* in the network and the distance *L*_*ij*_ between them is the number of edges of the shortest path between them. For example, the shortest path between nodes “cat” and “she” is 2 in Fig 1. The longest path among all minimum paths represents the diameter of the network. For example, the diameter of the network in Fig 1 is 13—that is, the path between “an” and “jumped.”

If a network has a small Average Path Length <*l*> (<*l*> ~ <*l*_*rnd*_>), and a large Clustering Coefficient (*C*), then we can call it a Small World network. However, this definition is rather qualitative, because it is difficult to decide whether the observed value is small or large. In general, it requires comparing the observed <*l*> and (*C*) to that of a stochastic network. The stochastic network model used in this paper is the ER model proposed by Erdös and Renyi [55]. Humphries & Gurney defined a quantitative parameter, the *S*-Small-worldness, for measuring the Small World feature [56]. The formula is as follows:

$$S=\frac{C/C_{rnd}}{l/l_{rnd}}$$

In this formula, *C* denotes the network Clustering Coefficient, *C*_*rnd*_ represents the Clustering Coefficient of the ER random network with the same number of nodes and the same Average Degree; *l* denotes the Average Minimum Path, and *l*_*rnd*_ represents the Average Path Length of the ER random network. When the *S* is greater than *1*, the network can be considered as a Small World network.

#### Degree-dependent clustering coefficient and modularity

The degree-dependent clustering coefficient $\bar{C}\left(k\right)$ actually reflects the influence of the node to its neighboring nodes’ clustering coefficients. $\bar{C}\left(k\right)$ is the mean of the clustering coefficients of all nodes with degree *k*. If the degree *k* is represented by the X-axis, $\bar{C}\left(k\right)$ denotes the Y-axis, and the data distribution conforms to the power law, then the network is a hierarchical network. Again, as with many networks in the real world, language complex networks’ $\bar{C}\left(k\right)$ distributions usually meet this condition, i.e., $\bar{C}\left(k\right)\sim k^{-\gamma }$. This means that language complex networks tend to present as hierarchical structures. In short, the so-called hierarchical structure refers to such a network model: nodes with low degrees tend to form relatively dense sub-networks, the connections between these sub-networks are rather sparse, and then the nodes with high degrees will connect these sub-networks into a large connected network [16].

Modularity is a parameter that is closely related to the hierarchy feature of networks. It is generally believed that hierarchical networks will show modular characteristics and that these two are interrelated [57]. Modularity, in fact, is a parameter that concerns the clusters or communities in networks. However, a widely recognized, unique definition of the “community structure” within a network is still absent. The commonly used definition is based on the relative frequency of links: the nodes in a network can be divided into groups, and the links within a group are thought to be dense while the links between groups are sparser. In this definition, the criteria for “dense” and “sparse” are unclear, thus limiting the usefulness of this definition. Therefore, researchers have tried to offer a more quantitative definition of this concept. Newman proposed the *Q*-modularity index for measuring the modularity feature of networks, and this is the most commonly accepted method nowadays [58]. It is also the method we use for analyzing modularity of language networks in this study.

#### Other indicators

The Mean Length of Utterance (MLU), which is often treated as one of network parameters, is also observed. The definition of utterance is in Section “Word co-occurrence networks.”

The Average Degree *k* is the average of all nodes’ degrees.

Density refers to the ratio of the number of existing edges to theoretical maximum number of edges that one network, with the same number of nodes, can have. It reflects how dense a network is. The four observed language networks in this study are rather sparse networks [59], which indicates the low probability of connections between two random words.

The Centrality of a node can reveal its importance in the network. We use the most common centrality indicators in this work: Degree Centrality and Betweenness Centrality. Degree Centrality is the degree of a node. Obviously, this indicator only considers the direct neighbors of a node, so it reflects only the local importance of the node, while the Betweenness Centrality of a node is more focusing on the macro importance. If a node were on many shortest paths of other node-pairs, it would occupy a relatively central position in the network. Since the Betweenness Centrality considers not only the direct neighbors of a node, but also the other nodes in the network, it can reflect the overall importance of a node in the network better.

The Centrality of the whole network is generally called Centralization. Degree Centralization reflects how strong the local hubs (central nodes) are in a network. In language networks, the Degree Centralization reveals the strength of the local hubs (high-degree nodes). The greater the Degree Centralization of a network, the stronger the combination abilities of high-degree nodes/words in the network.

## Results

Hierarchy is also recognized to be a result of other features. It is generally believed that hierarchical networks must first share some characteristics with complex networks (such as being small-world and scale-free); furthermore, the hierarchicality is likely to be closely related to the origin of these complex network features. For investigating the evolution process of Chinese networks, we will observe and describe the topological characteristics (including small-world and scale-free), the modularity, and the hierarchicality of four diachronic Chinese networks. The relationships between these networks characteristics and some common linguistic features, such as the frequency and length distributions of linguistic units, are also discussed. The observed network parameters, *n* = number of nodes; <*k*> = average degree; *P* = network density; *<C*_*rnd*_*>* = clustering coefficient of the random network counterpart; *<l>* = average path length; *<l*_*rnd*_*>* = average path length of the random network counterpart; *D* = diameter; *<C>* = clustering coefficient; *γ* = exponent of the power law that best fits to the degree distribution; *R*_*1*_^*2*^ = determination coefficient of the power law with exponent *γ*; *β* = exponent of the power law that best fits to ${\bar{K}}_{n}{}_{n}\left(k\right)$; *R*_*2*_^*2*^ = determination coefficient of the powerlaw with exponent *β*; *α* = exponent of the power law that best fits to degree dependent clustering coefficient $\langle \bar{C}\left(k\right)\rangle$; *δ* = coefficient of the the power law that best fits to $\langle \bar{C}\left(k\right)\rangle$; *R*_*3*_^*2*^ = determination coefficient of the power law with exponent *α*; *NC* = network centralization, are shown in Table 2. Corresponding random network parameters as a baseline are also presented in Table 2 with a tag ‘_*rnd*_’. To be specific, the random networks share the same numbers of nodes and average degrees with the observed networks. The *γ*, *β*, and *R*^*2*^ parameters are absent for the random networks since their degree distributions and average degree of nearest neighbors distributions do not fit to power law functions.

**Table 2:** Diachronic change of network parameters.

Parameters	Network 1	Network 2	Network 3	Network 4
*N*	1,527	3,034	2,227	2,526
*<k>*	6.6051	4.2149	5.1612	4.7403
*<k*_*rnd*_*>*	6.6064	4.2373	5.1863	4.7593
*P*	0.0022	0.0007	0.0012	0.0020
*P*_*rnd*_	0.0043	0.0014	0.0023	0.0019
*<l>*	3.6553	4.2421	4.3153	4.7460
*<l*_*rnd*_*>*	4.0943	5.6825	4.8679	5.1763
*D*	9	13	14	16
*D*_*rnd*_	7	13	9	10
*<C>*	0.1542	0.0716	0.0681	0.0563
*<C*_*rnd*_*>*	0.0061	0.0016	0.0028	0.0013
*NC*	0.246	0.163	0.107	0.199
*NC*_*rnd*_	0.0093	0.0109	0.0131	0.0088
*Q*-modularity measure	0.3481	0.4846	0.4345	0.4832
*Q*_*rnd*_	0.3935	0.5152	0.4522	0.4768
*S* (Small-worldness measure)	35.9567	57.6239	27.5611	28.2322
*γ*	-1.173	-1.270	-1.353	-1.449
*R*_*1*_^*2*^	0.775	0.811	0.851	0.824
*β*	-0.418	-0.396	-0.185	-0.355
*R*_*2*_^*2*^	0.835	0.729	0.566	0.661
*δ*	0.956	0.454	0.304	0.292
*δ*_*rnd*_	5.797	3.966	5.652	4.229
*α*	-0.609	-0.576	-0.375	-0.543
*α*_*rnd*_	-2.543	-2.262	-2.5	-2.317
*R*_*3*_^*2*^	0.803	0.688	0.564	0.571
*R*_*3*_^*2*^_*rnd*_	*0*.*9986*	0.9951	0.9996	0.9996

A MANOVA test (Model as independent variable, Time as concomitant variable, and *P*, <*l*>, *D*, <*C*>, *NC*, *Q*-modularity measure, *δ*, *α*, *R*_*3*_^*2*^ as dependent variables) shows that there is a significant difference between two models with *P*≈0.033<0.05. We discuss the details on change of co-occurrence networks from several different aspects below.

### Evolution of degree distribution

Overall, in a co-occurrence network, the degree of a node/word reflects how many other words it can be adjacent to in sentences. Meara has referred to this co-occurrence relationship as co-citation [50]. Linguistically speaking, the degree of a word can be explained, in a way, as a word’s “valence,” which usually means the syntactic ability of a word—usually a verb though sometimes an adjective or a noun—to combine with complements or adjuncts [60].

In this paper, the interpretation of the valence is based on PVP theory (Probabilistic Valency Pattern Theory) [61, 62]. Unlike the common definition of the term, the distinction between complements and adjuncts [60] is ignored in PVP; therefore, valence is regarded as a general syntactic capacity of a linguistic unit (word) combining with other linguistic units (words). In language networks, the valence of a word is equal to the sum of its centripetal (input) and centrifugal (output) degrees. The centripetal degree refers to the ability of a word to be dominated by other words, while the centrifugal degree refers to the ability of a word to dominate other words. Since the study is based on word co-occurrence networks, the parameter “degree” refers to the ability of words to be adjacent to other words in sentences. Since about 50% of the syntactic dependencies are found between linearly adjacent words in Chinese sentences [63], word co-occurrence networks in this study could reflect, to some extent, the syntactic patterns of Chinese.

**Fig 2:**
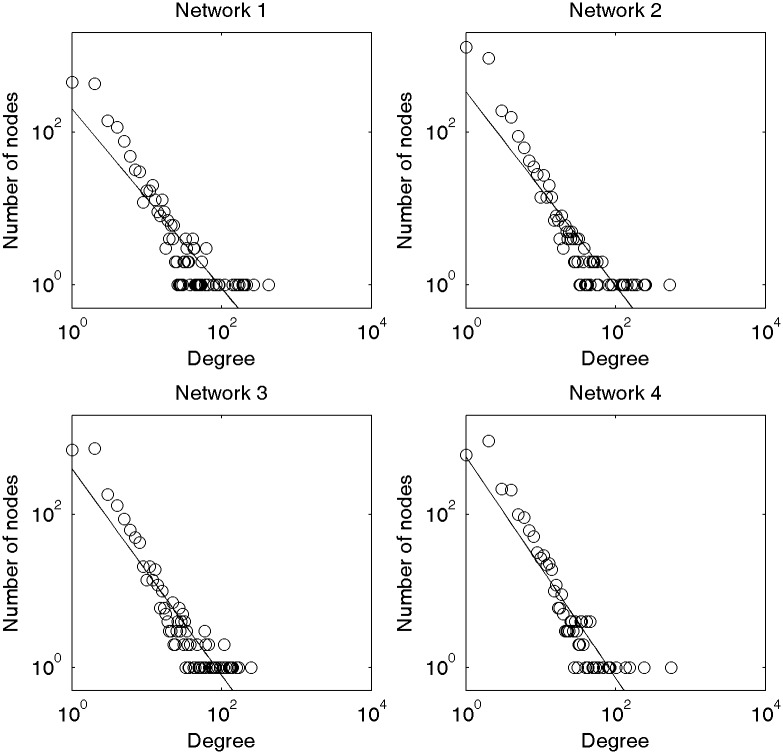
Power-law fitting (dual-log) of degree distributions of four diachronic Chinese networks.

Fig 2 shows the results of fitting the degree distributions to the power law.

According to Table 2, the absolute value of the exponential *γ* of the four power law distributions is increasing, which indicates that the enhancement of the power law of the degree distribution. In other words, few high-degree nodes are seeing increases, or the degrees of most of the nodes are experiencing reductions.

Since the degree distributions fit to the power law, observed networks are all scale-free networks. The scale-free feature of networks reveals that only a very small number of nodes/words have a strong ability to combine with other nodes/words, and most of the nodes/words have a weak capacity. This phenomenon can be explained by the “principle of least effort” [11]. The ability of a word to combine with other words is in proportion to its flexibility of use. Only a limited number of words have high flexibility, a quality which allows them to be used in a rich variety of contexts; their high frequency of use facilitates communication for both speakers/writers and listeners/readers. To be more specific, a small number of contextually flexible words can reduce the encoding effort for speakers/writers, and the remaining words that are usually used in a non-ambiguous context can reduce the decoding effort for listeners/readers. Therefore, the two parts work together and achieve a dynamic balance.

By observing the change of the parameter *γ*, we can see that the power law distribution of the Chinese word co-occurrence networks is becoming stronger over time. It means that the binding ability of a small number of words is increasing, and more and more words have a relatively weak capacity. The scale-free network is also called an authoritarian network, and from this point of view, the Chinese network has become more authoritarian over time.

### Evolution of the average degree of nearest neighbors

**Fig 3:**
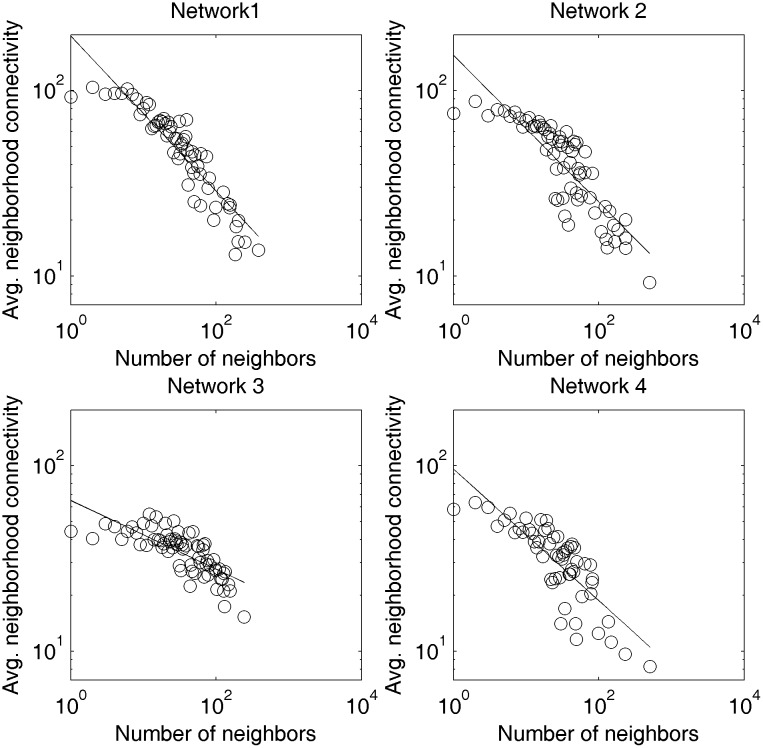
Power-law fitting (dual-log) of average degree of nearest neighbors of four diachronic Chinese networks.

The average degree of nearest neighbors, ${\bar{K}}_{n}{}_{n}\left(k\right)$, reflects the influence of one node’s degree on its neighbors’ degrees. It represents the correlation between the degree of a node and the degrees of its neighbors. Complex language networks tend to show a clear degree of correlation. Dynamic language networks often exhibit negative correlations, which means that language units with strong (weak) binding abilities tend to be combined with nodes with weak (strong) binding capabilities. Fig 3 shows the linear fitting of the ${\bar{K}}_{n}{}_{n}\left(k\right)$ of the four Chinese networks.

In Fig 3, the X-axis represents the degrees and the Y-axis represents the average degrees of nodes which are neighbors of nodes with certain degree. It can be seen from Fig 3 that there has been a negative correlation between these two values for all four diachronic Chinese networks. Therefore, a high-degree node (a word with a strong combination capacity) tends to link to low-degree nodes (a word with a weak combination ability). However, the change is not one-directional. The absolute value of the slope decreased from periods 1 to 3, while it increased slightly in period 4.

### Evolution of the small-world feature

**Fig 4:**
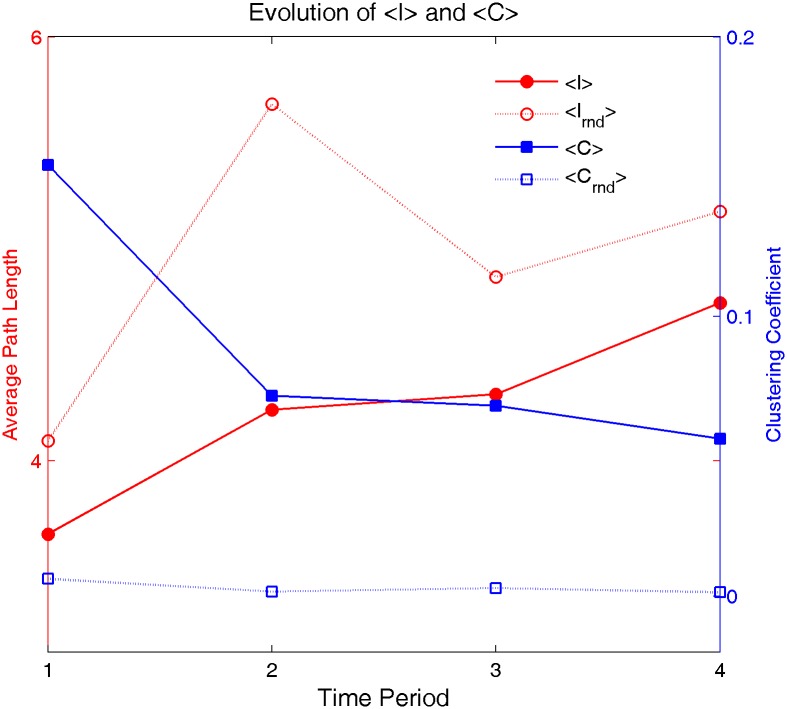
Evolution of the clustering coefficient (*<C>*) and the average path length (*<l>*).

According to the model of Watts and Strogatz [54], small-world networks can be defined by two complex network parameters—namely, the clustering coefficient *<C>* and the average path length (*<l>*). The evolution of the average path length (or characteristic path length) is shown in Fig 4. According to the *S* value in Table 2, these four networks are all small-world networks. However, from Fig 4 we can see that although *<C>* is continuously getting smaller, *<l>* is increasing over time. This means that the Chinese word co-occurrence network is expanding although it is still a small-world network.

Meara has suggested that clustering seems to be a fundamental attribute of words in a text and may be related to the repetition of some words in the text [50]. It is also believed that the structure of a complex network affects the functionality of a system [64]. Studies have shown that small-world networks can provide efficient communication between nodes [65, 66].

According to Fig 4, the average path length showed an increasing trend, while the clustering coefficient showed a decreasing tendency. A MANOVA test (Model as independent variable, Time as concomitant variable, and <*C*>, <*l*> as dependent variable) shows that there is a significant difference between two models with *P*≈0.035<0.05. This means that although the Chinese word co-occurrence network maintains as a small-world network, it has become increasingly larger globally, but the local connection has become increasing weaker.

### Evolution of the degree-dependent clustering coefficients

Hierarchy is the core organizational principle of complex networks and it is also the best observation spot for exploring the phenomenon of networks [15]. Ravasz, Somera, Mongru, Oltvai and Barabási proposed using the degree-dependent clustering coefficient as a verification index for the network’s hierarchy feature, which is also a commonly accepted method in most language network studies [16]. Hierarchical networks are characterized by the tendency of low-degree nodes to form tightly connected subnets, with the high-degree nodes then linking these subnets into a connected system.

**Fig 5:**
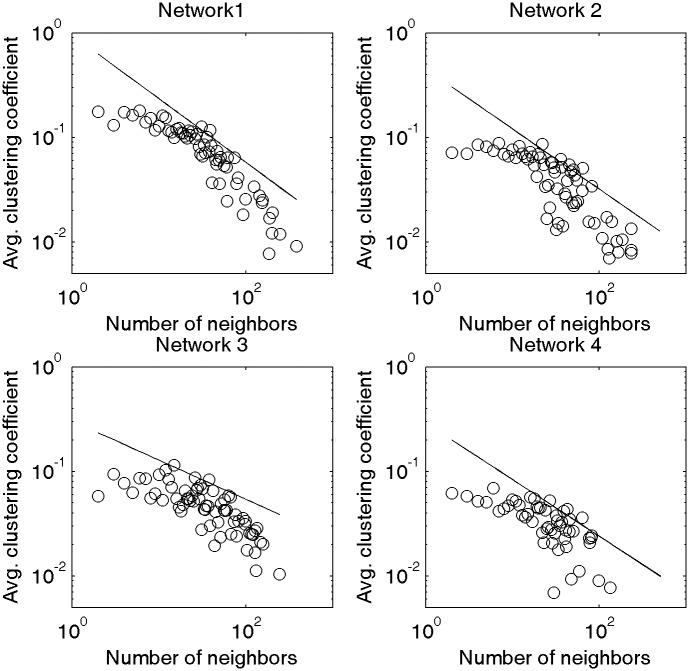
Power-law fitting of the degree-dependent clustering coefficients of four diachronic Chinese networks.

Fig 5 shows the power-law fitting results for the degree-dependent clustering coefficients of the four Chinese networks. The results show that all four networks exhibit hierarchical structures, which means that the words with weak combination capacity tend to form small groups first, and the words with strong combination capacity connect them into a whole.

These four distributions of the degree-dependent clustering coefficients are very similar to those of the Fig 3 in the [57], all of which belong to the hierarchical network. In these four diachronic language networks, $\bar{C}\left(k\right)$ decreases as parameter *k* increases. This illustrates the existence of hierarchical structures, which means that low-degree nodes are more likely to form small groups with a high density, and these small groups are weakly connected. Meanwhile, the high-degree nodes (usually representing functional words) connect these small groups into a whole. From the diachronic point of view, the degree of fit shows a decreasing trend.

**Fig 6:**
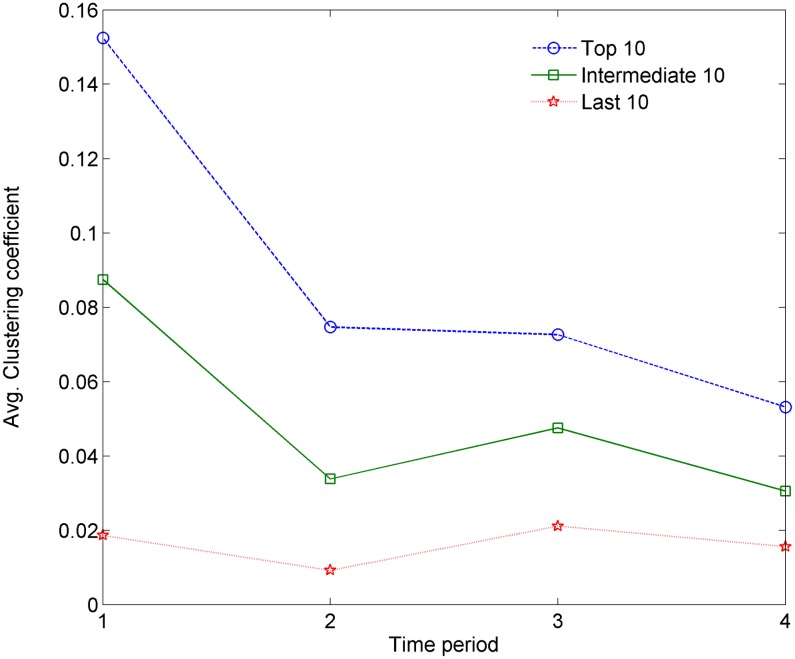
The means of the top 10, the middle 10, and the final 10 degree-dependent clustering coefficients.

In order to further investigate the difference between the degree-dependent clustering coefficients of nodes with different degrees, we calculate the means of the top 10, the middle 10 and the lowest 10 degree-dependent clustering coefficients for the four networks. The results are shown in Fig 6. It can be seen that there is no significant change for the average degree-dependent clustering coefficient of the top 10 high-degree nodes; it maintains the same level. There is an obvious decrease from period 1 to period 2 for the average degree-dependent clustering coefficient of the middle 10 high-degree nodes; however, it becomes more stable for the remaining periods. There is an observed degreasing tendency for the average degree-dependent clustering coefficient of the last 10 high-degree nodes. The evolution of the hierarchy is closely related to the distribution of node degrees and degree dependent parameters, which we will discuss more in detail later.

Modularity is a concept that is closely related to hierarchicality. Barabási, Ravasz and Oltvai argued that hierarchical organization and modularity are the most important organizational principles for many complex networks, and it is often assumed that the module structure and the hierarchical structure are usually found together in a complex network [57]. Many real world networks are hierarchical, such as www networks and biological networks, which generally have both scale-free and community structures [16]. Such communities are also called modules. Sporns, Chialvo, Kaiser and Hilgetag argued that modular networks are small-world networks [67]. On the one hand, the high clustering of nodes in the same module can ensure efficiency in completing specific functions, such as visual dynamic tracking in biological networks; on the other hand, the small average path length in a macro sense can ensure that the system can complete some more general functions, such as working memory.

In fact, the degree of modularity refers to the difference between a network with a community structure and a random network, since a random network lacks a community structure. The greater the difference, the more significant the community is. Newman proposed the following modularity calculation method: because the modularity of a random network is less than 0.3, a network with a modularity greater than 0.3 has a clear community structure [58]. As can be seen from the Table 2, *Q*-modularity values of four networks are all greater than 0.3, so there is a clear module structure in all of them. Furthermore, the value of *Q* has also evolved. Overall, the *Q* is increasing over time.

### Evolution of mean utterance length and mean word length

The change of the Chinese word co-occurrence network is accompanied by the change of the mean utterance length (MUL) and mean word length (MWL).

Although MUL is not a network parameter, it is generally regarded as one in language network research. The reason for this is mainly that the construction of the network is closely related with the UL, no matter whether the network is based on dependency syntax or word co-occurrence relationships. Cross-sentence relationships are not allowed. In this paper, an utterance is defined as a “chunk,”—that is, a continuous word string. It is similar to a clause in grammar.

**Fig 7:**
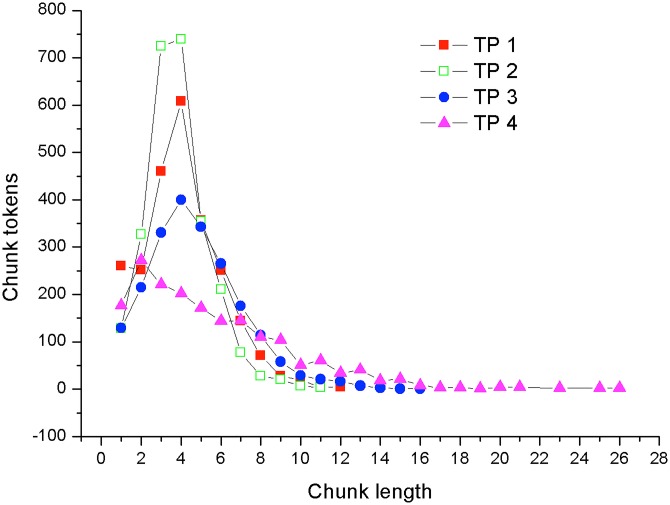
MUL distributions (TP1 corresponds to Network 1 text).

Fig 7 illustrates the four MUL distributions. The X-axis represents the length of the utterances, and the Y-axis represents the number of utterances sharing the same length.

It can be seen from Fig 7 that the distribution does not change significantly from period 1 to period 2; however, it becomes broader in period 3, and it continues the same broadening tendency in period 4. In addition, the distribution is obviously right-skewed, and the maximum number of utterances lessens.

In addition to the increasing of the MUL, the MWL of texts also increases. This means that the MUL of Chinese shows an increasing trend no matter whether it is measured based on words or Chinese characters. In Table 3, the WL (static), measured based on word types, and the WL (dynamic), measured based on tokens, are both increasing over time.

**Table 3:** The diachronic change of the MUL of Chinese (measured based on words) and the MWL of Chinese (measured based on characters).

	TP 1	TP 2	TP 3	TP 4
MUL	4.07	3.82	4.74	5.55
MWL(static)	1.17	1.26	1.35	1.79
MWL(dynamic)	1.48	1.66	1.71	2.01

## Discussion

Language is a complex adaptive system, and the more ancient a language is, the more complex it may be [14], but how does a language become complex? In this paper, Chinese is used as a means for investigating this question from the perspective of hierarchical network systems. We built four diachronic word co-occurrence networks based on texts of ancient Chinese, middle ancient times Chinese, modern times Chinese, and modern Chinese, and then analyzed the topological changes of these networks over time.

The questions that we address first are whether Chinese word co-occurrence networks show any complex characteristics and whether network parameters have changed over time. According to our observations, all four diachronic Chinese word co-occurrence networks show similar complex network characteristics, such as scale-free and small-world features. However, they are different in terms of fine details. More specifically, the power law fitted distributions of words’ degrees have become extreme. This means that the high-degree words (they are usually also the central words, such as functional words) are developing stronger combination capacity compared to other words, while most other words have a weak ability to combine with other words. Since the average clustering coefficient <*C*> of networks has declined, it can be inferred that the connections between low-degree words are also diminishing. The small-world feature of networks ensures the efficiency of communication. Judging from observed data, although the four diachronic networks all show the small-world feature, the evolution of the average clustering coefficients and the average path lengths, two parameters related to the small-world feature, shows that the language network is actually getting larger. This means that the language system is becoming more complex in the lexical network sense. Moreover, the enhancement of the scale-free feature means that people are, consciously or unconsciously, adhering to the “Principal of Least Effort” more and more, so that they can avoid the increasing difficulty of communicating along with the growing of the language network [68].

The average degree of nearest neighbors is also an important network. Previous studies have confirmed that dynamic language networks show significant negative correlations with this feature, which means that low-degree words are more likely to be connected with high-degree central words. According to our findings, this negative correlation decreased from periods 1 to 3, meaning that high-degree words were beginning to connect more often with medium-degree words. Low-degree words were doing so as well. However, this trend rebounded from periods 3 to 4, although the negative correlation was still weaker than in periods 1 and 2. We find that the changing tendency of the negative correlation is consistent with the change of the network centrality (see *NC* in Table 2) and the betweenness (see **SI**.). In fact, the average degree of nearest neighbors is closely related to the centrality of nodes. For example, Ferrer-i-Cancho and Solé found out that the average degree of nearest neighbors decreases after removing central nodes from the language networks [30].

Regarding the hierarchy features of language networks (i.e., the degree-dependent clustering coefficient), the four word co-occurrence networks all show similar distributions. Clustering coefficients of nodes decreased in average with the increase of the node degree, which means that the Chinese word co-occurrence network has consistently hierarchical characteristics. Low-degree words with weak combination capacities have tended to form tightly connected sub-lexical groups, and then the high-degree words, which are usually functional words, have linked these sub-lexical groups into a whole. However, over time, the degree-dependent clustering coefficient has decreased. The reason for this is that the degree-dependent clustering coefficients of low-degree words are decreasing continuously, while those of high-degree words and medium-degree words do not change significantly. It is generally believed that network hierarchy is accompanied by its modularity. The results of this study show that the modularity of all four diachronic networks is greater than 0.3, indicating that they all have clear modular structures. However, from period 1 to periods 2, 3 and 4, the modularity of the Chinese word co-occurrence network generally increased, which shows the deepening trend of the communitization of Chinese vocabulary networks.

In synthesizing the above analyses, we can generally say that the number of the sub-lexical groups of Chinese word co-occurrence network is increasing over time. The high-degree words in the network remain the central nodes of the entire network. They are crucial and they maintain the small-world and scale-free features of the network, which ensure the efficiency of language communication. Meanwhile, the modularity of low-degree words is increasing, which indicates that the subgroups are becoming specialized and their number is increasing. Because of this specialization, they are more inclined to connect with medium-degree words with which they share some similarities, and then finally connect into a whole through the high-degree words. Interestingly, Borodkin, Kenett, Faust and Mashal [21] considered the average clustering coefficient as a parameter of local communitization, modularity as the parameter of communitization for mediators, and the average path length as the parameter of macro-scale communitization in networks. He found out lexical networks of the second language displayed greater local connectivity and less modular community structure than the network in the native language. This shows that if we see the language evolution from the point of view of its complexity, the increasing modularity of networks may actually mean higher language complexity. The study we present verifies this point with authentic diachronic data.

During its evolution, the Chinese word co-occurrence network experienced some changes while keeping its basic characteristics, including its small-world and scale-free qualities, the negative correlation of the average degree of nearest neighbors, and most importantly, the hierarchical feature. In general, the evolution of the Chinese word co-occurrence network shows the characteristics of stability and self-adaptation. As a complex adaptive system, the evolution of the Chinese word co-occurrence network, while having its own internal evolution as a dynamic symbol system, is inseparable from external social and cultural factors, which will be discussed in more detail below.

The relationship between the linear and hierarchical structures is also a question examined in this paper. Since the networks are based on word co-occurrence relationships, their sub-structures are closely related to the chunks (defined in section 2) and the distribution of the length of utterances (i.e., MUL). MUL can also be used as a rough measurement of syntactic complexity. The greater the MUL, the more complex the syntax. Over time, MUL increases, and in modern Chinese (network 4), it shows significant growth. In the networks, the parameter average path length is very closely related to the MUL because utterances are actually linear words sequences. Therefore, the length of utterances affects the length of the paths between two words. Jiang and Liu examined the relationship between sentence length and average dependency distance in Chinese and found that there is a positive correlation between these two parameters—that is, the longer the sentence, the greater the average dependency distance [69]. However, grammatical factors and cognitive factors such as memory limit the growth of the average dependent distance, making it as short as possible. This phenomenon is referred to as “minimizing dependency distance.” Although the word co-occurrence relationship we observed in this study is different from dependent syntax, they are indeed very similar. Approximately half of the dependency relationships are found between linearly adjacent words. In some ways, the average dependency distance and the average path length are similar [62]. According to our observations, the average path length also grows with the increase of MUL, but much more slowly. This may due to the influence of various human cognitive factors (e.g., short-term memory) on the evolution of language, especially in terms of grammar. For example, studies have shown that no more than seven blocks of information can be temporarily stored in the brain at any given time [70]. This may also mean that although Chinese has a hierarchical feature, it is developing more linear serialization and the word order of Chinese is also becoming more flexible. In addition, the reason why the average path length has maintained relatively steady growth may also be due to the emergence of some word collocation patterns [71, 72], which are very similar to the motivation for dependency minimization [69].

In fact, the increase in MUL is not only expressed in the number of words, but also by the average length of words, which is also growing (see Table 3). From the perspective of language information load, a higher MLU or word length usually indicates a higher level of language proficiency [73, 74]. Moreover, the growth is closely related to the need for more nuanced language expressions. As society develops, new things and new concepts appear. Language communication and expression become more refined in order to meet the need to communicate information. However, efficiency is also a fundamental principle of language communication. The longer the utterances and the words, the greater the cognitive complexity. For balancing these two needs, some syntactic changes are inevitable. The emergence of new word collocation patterns is likely to be the result of this adaptation.

By analyzing four diachronic Chinese word co-occurrence networks, we speculate the evolution mechanism was as follows: Initially, low-degree words, which are usually low-frequency words, formed into dense subgroups. Some high-degree words or hubs, usually function words, then linked these subgroups into a whole for ensuring the connection of the entire language network. Such a network is a very efficient organizational system. It ensures both the adequacy of the description and the efficiency of expression. As human society developed, people needed to find new words and syntactic structures to fit their changing world. Word and utterance length began to grow; the sub-lexical groups also became more specialized. This led to the increase of the width and length of the average path length. The small world became bigger. At the same time, the hierarchy feature of the network underwent a corresponding change. Due to the specialization of the sub-lexical groups, the connections between these low-degree words became less. Therefore, the clustering coefficient got smaller. The clustering coefficient of high-degree words remained similar, while that of the medium-degree words was reduced from period 1 to 2, and then remained at the same level. All these indicate that the modularity of the language network continues to develop. For dealing with the dilemma of increasing language network, people started to make some syntactic adjustments—that is, new word collocation patterns and more flexible word orders. That may be also the main reason for the observed phenomenon that the language network persisted as a small-world and scale-free network. It was to ensure the efficiency and to reduce the efforts of language communication. However, speculation regarding new word collocation patterns and more flexible word order requires further empirical investigation.

## Conclusion

This study constructed four diachronic word co-occurrence networks based on texts of ancient Chinese, middle ancient times Chinese, modern times Chinese, and modern Chinese, and investigated the evolution of Chinese as a hierarchical system. We find the following:

1. Hierarchy (based on the definition of network topology) exists in four diachronic Chinese networks, indicating that Chinese has always been a hierarchical complex adaptive system.
2. If the Chinese hierarchical system is divided into low (local groups), middle (middle layer clusters or small communities) and high (global structure) levels, then the evolution of Chinese (syntactic) network can be described as a) the connections of words at the micro-language level are continually weakening; b) the number of meso-level word communities had a significant growth in middle ancient times (network 2), and then maintained at a relatively high level; and c) the global network in the macro level is expanding. This means that more and more low-degree words tend to be connected to the medium-connection words and then the high-connection words will link these groups into a highly efficient small-world language network.
3. As the number of sub-words-communities as well as modularity increases, the written Chinese word co-ocurrence network becomes larger, which is consistent with the increase of the length of linear sequences, as measured by mean word length and mean utterance length. The emergence of new word collocation patterns and more flexible word orders may be important reasons for the language system to maintain its hierarchical structure.
4. We summarize the diachronic language developing mechanism of written Chinese word co-occurence network based on observed changes. With the development of human society, new things and new concepts emerged. There was a growing need for refined language expressions. The words of specific fields began to form either large or small sub-lexical groups. Words, clauses, and sentences also began to lengthen. For balancing the increase of language units and the efficiency of language communications, new word collocation patterns started to appear and word orders became more flexible. The language system could then offer more refined language expressions while at the same time achieving efficient language communication.

This paper investigates the evolution of written Chinese lexical networks spanning more than 2,000 years. Based on the co-occurrence of words in sentences, we have generated maps of the lexical structure of the texts. As a result, after probing into the changes of indicator values of the networks that are closely related to the hierarchy feature, we have found some diachronic patterns in the language network. This research brings new insights to understanding the emergence, adaptability, and stability of human language system.

## Supporting information

S1 File S1 Supporting information. (DOCX)
