# Supplementary material for: How does language change as a lexical network? An investigation based on written Chinese word co-occurrence networks
Source: PLoS One. 2018 Feb 28;13(2):e0192545. doi: 10.1371/journal.pone.0192545 (PMC5830315; doi:10.1371/journal.pone.0192545)
Supplement: S1 File — (DOCX) [file pone.0192545.s001.docx]

**How does language change as a lexical network?**

**An investigation based on written Chinese word co-occurrence networks**

**Supporting Information**

Heng Chen^a^, Xinying Chen^b^, Haitao Liu ^a, c,d^[[1]](#footnote-1)^*^

a Centre for Linguistics and Applied Linguistics, Guangdong University of Foreign Studies, Guangzhou, 510420, China

b School of Foreign Studies, Xi’an Jiaotong University, Xi’an, 710000, China

c Department of Linguistics, Zhejiang University, Hangzhou, 310058, China

d Ningbo Institute of Technology, Zhejiang University, Ningbo, 315100, China

1. **The English paragraph as a network construction example in section 2.2.**

“An old woman had a cat. The cat was very old; she could not run quickly, and she could not bite, because she was so old. One day the old cat saw a mouse; she jumped and caught the mouse. But she could not bite it; so the mouse got out of her mouth and ran away, because the cat could not bite it.”

1. **Degree distributions**

| Network 1 | | Network 2 | | Network 3 | | Network 4 | |
| --- | --- | --- | --- | --- | --- | --- | --- |
| Degree | Number of nodes | Degree | Number of nodes | Degree | Number of nodes | Degree | Number of nodes |
| 1 | 444 | 1 | 1291 | 1 | 705 | 1 | 605 |
| 2 | 427 | 2 | 926 | 2 | 736 | 2 | 917 |
| 3 | 141 | 3 | 191 | 3 | 183 | 3 | 216 |
| 4 | 115 | 4 | 157 | 4 | 131 | 4 | 211 |
| 5 | 76 | 5 | 88 | 5 | 87 | 5 | 100 |
| 6 | 48 | 6 | 62 | 6 | 63 | 6 | 91 |
| 7 | 32 | 7 | 42 | 7 | 50 | 7 | 62 |
| 8 | 30 | 8 | 35 | 8 | 43 | 8 | 52 |
| 9 | 12 | 9 | 28 | 9 | 21 | 9 | 32 |
| 10 | 17 | 10 | 14 | 10 | 14 | 10 | 27 |
| 11 | 17 | 11 | 27 | 11 | 21 | 11 | 29 |
| 12 | 20 | 12 | 14 | 12 | 14 | 12 | 22 |
| 13 | 13 | 13 | 20 | 13 | 19 | 13 | 23 |
| 14 | 9 | 14 | 14 | 14 | 12 | 14 | 19 |
| 15 | 8 | 15 | 7 | 15 | 6 | 15 | 10 |
| 16 | 13 | 16 | 8 | 16 | 10 | 16 | 12 |
| 17 | 9 | 17 | 7 | 17 | 6 | 17 | 6 |
| 18 | 3 | 18 | 4 | 18 | 5 | 18 | 6 |
| 19 | 7 | 19 | 8 | 19 | 4 | 19 | 9 |
| 20 | 4 | 20 | 3 | 20 | 3 | 20 | 5 |
| 21 | 6 | 21 | 6 | 21 | 3 | 21 | 3 |
| 22 | 4 | 22 | 5 | 22 | 7 | 22 | 3 |
| 23 | 6 | 23 | 4 | 23 | 2 | 23 | 3 |
| 24 | 2 | 24 | 5 | 24 | 2 | 24 | 3 |
| 25 | 2 | 25 | 4 | 25 | 3 | 25 | 4 |
| 26 | 1 | 26 | 5 | 26 | 4 | 26 | 4 |
| 27 | 1 | 27 | 4 | 27 | 6 | 27 | 3 |
| 28 | 1 | 28 | 2 | 28 | 3 | 28 | 1 |
| 29 | 1 | 29 | 2 | 29 | 4 | 29 | 4 |
| 30 | 1 | 30 | 4 | 30 | 5 | 30 | 3 |
| 31 | 2 | 31 | 2 | 31 | 2 | 31 | 1 |
| 32 | 2 | 32 | 4 | 32 | 4 | 32 | 2 |
| 33 | 4 | 33 | 1 | 33 | 1 | 33 | 2 |
| 34 | 3 | 34 | 1 | 34 | 3 | 34 | 4 |
| 35 | 2 | 37 | 2 | 35 | 2 | 35 | 4 |
| 36 | 2 | 38 | 3 | 36 | 1 | 37 | 2 |
| 38 | 1 | 39 | 1 | 37 | 1 | 40 | 1 |
| 41 | 4 | 40 | 1 | 38 | 2 | 41 | 4 |
| 42 | 3 | 41 | 1 | 42 | 1 | 42 | 1 |
| 43 | 3 | 42 | 1 | 44 | 1 | 43 | 1 |
| 44 | 1 | 45 | 2 | 47 | 2 | 46 | 4 |
| 46 | 1 | 46 | 1 | 49 | 1 | 49 | 1 |
| 47 | 1 | 49 | 2 | 50 | 1 | 50 | 1 |
| 48 | 1 | 51 | 2 | 52 | 1 | 51 | 1 |
| 49 | 1 | 52 | 2 | 55 | 1 | 54 | 1 |
| 50 | 1 | 56 | 1 | 58 | 1 | 59 | 1 |
| 52 | 1 | 57 | 2 | 59 | 3 | 68 | 1 |
| 53 | 1 | 58 | 1 | 60 | 1 | 80 | 1 |
| 54 | 2 | 66 | 2 | 61 | 2 | 81 | 1 |
| 60 | 1 | 80 | 1 | 67 | 2 | 83 | 1 |
| 62 | 3 | 87 | 1 | 68 | 1 | 84 | 1 |
| 66 | 1 | 91 | 1 | 74 | 1 | 101 | 1 |
| 78 | 1 | 115 | 1 | 75 | 1 | 137 | 1 |
| 84 | 1 | 123 | 1 | 76 | 1 | 153 | 1 |
| 85 | 1 | 125 | 1 | 79 | 1 | 242 | 1 |
| 91 | 1 | 130 | 1 | 82 | 1 | 553 | 1 |
| 93 | 1 | 142 | 1 | 83 | 1 | - | - |
| 110 | 1 | 163 | 1 | 88 | 1 |  |  |
| 140 | 1 | 174 | 1 | 95 | 1 |  |  |
| 152 | 1 | 191 | 1 | 106 | 1 |  |  |
| 163 | 1 | 241 | 1 | 108 | 2 |  |  |
| 171 | 1 | 246 | 1 | 117 | 1 |  |  |
| 190 | 1 | 250 | 1 | 126 | 1 |  |  |
| 198 | 1 | 524 | 1 | 127 | 1 |  |  |
| 202 | 1 | - | - | 132 | 1 |  |  |
| 217 | 1 |  |  | 136 | 1 |  |  |
| 266 | 1 |  |  | 138 | 1 |  |  |
| 427 | 1 |  |  | 140 | 1 |  |  |
| - | - |  |  | 159 | 1 |  |  |
|  |  |  |  | 169 | 1 |  |  |
|  |  |  |  | 247 | 1 |  |  |

1. **Average degree of nearest neighbors**

| Network 1 | | Network 2 | | Network 3 | | Network 4 | |
| --- | --- | --- | --- | --- | --- | --- | --- |
| No. of neighbors | Avg. neighborhood connectivity | No. of neighbors | Avg. neighborhood connectivity | No. of neighbors | Avg. neighborhood connectivity | No. of neighbors | Avg. neighborhood connectivity |
| 1 | 92.55 | 1 | 75.46 | 1 | 44.30 | 1 | 58.13 |
| 2 | 103.83 | 2 | 87.62 | 2 | 40.56 | 2 | 63.30 |
| 3 | 95.78 | 3 | 73.22 | 3 | 48.77 | 3 | 59.74 |
| 4 | 96.46 | 4 | 78.84 | 4 | 46.92 | 4 | 47.32 |
| 5 | 96.38 | 5 | 77.80 | 5 | 40.11 | 5 | 50.84 |
| 6 | 102.10 | 6 | 72.78 | 6 | 44.20 | 6 | 55.28 |
| 7 | 95.33 | 7 | 76.63 | 7 | 46.75 | 7 | 43.74 |
| 8 | 90.05 | 8 | 70.88 | 8 | 43.43 | 8 | 45.89 |
| 9 | 74.20 | 9 | 63.65 | 9 | 37.67 | 9 | 43.84 |
| 10 | 79.86 | 10 | 69.07 | 10 | 48.87 | 10 | 52.25 |
| 11 | 86.06 | 11 | 71.35 | 11 | 37.19 | 11 | 45.29 |
| 12 | 84.12 | 12 | 63.69 | 12 | 54.81 | 12 | 43.28 |
| 13 | 62.33 | 13 | 64.62 | 13 | 47.59 | 13 | 39.03 |
| 14 | 64.30 | 14 | 64.11 | 14 | 39.87 | 14 | 36.11 |
| 15 | 64.99 | 15 | 67.74 | 15 | 52.87 | 15 | 38.85 |
| 16 | 67.48 | 16 | 64.83 | 16 | 39.96 | 16 | 51.28 |
| 17 | 68.76 | 17 | 63.04 | 17 | 39.56 | 17 | 32.46 |
| 18 | 68.03 | 18 | 61.76 | 18 | 36.24 | 18 | 44.99 |
| 19 | 70.75 | 19 | 47.92 | 19 | 37.97 | 19 | 50.56 |
| 20 | 66.18 | 20 | 56.48 | 20 | 48.85 | 20 | 45.96 |
| 21 | 57.07 | 21 | 58.76 | 21 | 42.33 | 21 | 37.96 |
| 22 | 67.58 | 22 | 64.61 | 22 | 34.55 | 22 | 24.43 |
| 23 | 59.97 | 23 | 54.83 | 23 | 38.93 | 23 | 23.41 |
| 24 | 63.96 | 24 | 45.65 | 24 | 40.03 | 24 | 41.15 |
| 26 | 46.42 | 25 | 26.14 | 25 | 36.07 | 25 | 24.60 |
| 27 | 55.21 | 26 | 37.77 | 26 | 50.34 | 26 | 32.96 |
| 29 | 55.00 | 27 | 25.70 | 27 | 40.23 | 27 | 41.44 |
| 30 | 43.02 | 28 | 53.13 | 28 | 39.38 | 28 | 32.25 |
| 31 | 68.21 | 29 | 56.20 | 29 | 37.55 | 29 | 24.86 |
| 32 | 45.69 | 30 | 50.63 | 30 | 44.18 | 30 | 14.10 |
| 33 | 50.83 | 31 | 53.00 | 31 | 28.89 | 31 | 31.44 |
| 34 | 51.85 | 32 | 26.25 | 32 | 38.73 | 32 | 34.00 |
| 36 | 54.35 | 33 | 38.39 | 33 | 27.24 | 33 | 33.00 |
| 38 | 56.48 | 34 | 21.06 | 34 | 37.62 | 34 | 16.97 |
| 39 | 69.69 | 36 | 60.08 | 35 | 35.68 | 35 | 34.79 |
| 40 | 48.03 | 37 | 49.32 | 38 | 37.09 | 37 | 36.01 |
| 41 | 30.95 | 38 | 18.89 | 42 | 43.52 | 39 | 26.08 |
| 45 | 44.16 | 40 | 40.90 | 44 | 22.39 | 40 | 26.03 |
| 46 | 38.59 | 41 | 29.80 | 45 | 29.20 | 41 | 36.63 |
| 47 | 46.81 | 43 | 47.09 | 47 | 26.94 | 42 | 27.62 |
| 49 | 35.96 | 45 | 53.29 | 48 | 44.15 | 43 | 37.77 |
| 50 | 25.12 | 47 | 51.19 | 51 | 26.29 | 45 | 26.77 |
| 51 | 44.94 | 49 | 28.10 | 55 | 37.10 | 46 | 36.15 |
| 56 | 39.07 | 50 | 52.46 | 57 | 36.69 | 48 | 14.08 |
| 60 | 35.36 | 51 | 25.82 | 58 | 31.89 | 49 | 11.61 |
| 61 | 23.93 | 52 | 37.94 | 60 | 30.00 | 50 | 30.35 |
| 62 | 45.65 | 54 | 35.06 | 66 | 35.21 | 59 | 19.71 |
| 74 | 44.04 | 55 | 35.88 | 67 | 37.37 | 64 | 29.64 |
| 78 | 29.78 | 57 | 27.12 | 68 | 25.12 | 77 | 29.23 |
| 81 | 33.63 | 64 | 36.20 | 70 | 38.01 | 78 | 20.46 |
| 93 | 20.00 | 65 | 46.75 | 73 | 29.51 | 81 | 23.51 |
| 99 | 23.49 | 76 | 26.45 | 76 | 30.87 | 82 | 24.56 |
| 125 | 28.26 | 82 | 35.83 | 78 | 25.78 | 99 | 12.47 |
| 144 | 24.55 | 88 | 21.89 | 82 | 29.21 | 135 | 14.40 |
| 153 | 23.34 | 108 | 17.30 | 93 | 31.06 | 148 | 11.18 |
| 156 | 24.13 | 122 | 23.69 | 98 | 27.71 | 230 | 9.65 |
| 184 | 13.09 | 125 | 15.75 | 99 | 27.18 | 506 | 8.25 |
| 188 | 18.45 | 130 | 14.22 | 103 | 21.52 | - | - |
| 201 | 15.29 | 140 | 22.30 | 110 | 27.74 |  |  |
| 204 | 19.88 | 159 | 18.76 | 117 | 24.54 |  |  |
| 249 | 15.22 | 166 | 15.33 | 121 | 24.44 |  |  |
| 381 | 13.78 | 184 | 17.76 | 127 | 21.17 |  |  |
| - | - | 233 | 20.09 | 128 | 26.24 |  |  |
|  |  | 234 | 15.98 | 131 | 17.43 |  |  |
|  |  | 235 | 14.15 | 133 | 26.87 |  |  |
|  |  | 497 | 9.22 | 148 | 22.89 |  |  |
|  |  | - | - | 156 | 21.11 |  |  |
|  |  |  |  | 243 | 15.30 |  |  |

1. **Degree-dependent clustering coefficients**

| Network 1 | | Network 2 | | Network 3 | | Network 4 | |
| --- | --- | --- | --- | --- | --- | --- | --- |
| No. of neighbors | Avg. clustering coefficient | No. of neighbors | Avg. clustering coefficient | No. of neighbors | Avg. clustering coefficient | No. of neighbors | Avg. clustering coefficient |
| 2 | 0.1764 | 2 | 0.0717 | 2 | 0.0581 | 2 | 0.0622 |
| 3 | 0.1310 | 3 | 0.0697 | 3 | 0.0941 | 3 | 0.0580 |
| 4 | 0.1744 | 4 | 0.0849 | 4 | 0.0776 | 4 | 0.0521 |
| 5 | 0.1640 | 5 | 0.0813 | 5 | 0.0625 | 5 | 0.0509 |
| 6 | 0.1800 | 6 | 0.0749 | 6 | 0.0860 | 6 | 0.0693 |
| 7 | 0.1398 | 7 | 0.0886 | 7 | 0.0852 | 7 | 0.0419 |
| 8 | 0.1524 | 8 | 0.0689 | 8 | 0.0556 | 8 | 0.0441 |
| 9 | 0.1172 | 9 | 0.0662 | 9 | 0.0611 | 9 | 0.0468 |
| 10 | 0.1285 | 10 | 0.0763 | 10 | 0.0930 | 10 | 0.0540 |
| 11 | 0.1612 | 11 | 0.0643 | 11 | 0.0530 | 11 | 0.0520 |
| 12 | 0.1553 | 12 | 0.0823 | 12 | 0.1025 | 12 | 0.0477 |
| 13 | 0.1161 | 13 | 0.0701 | 13 | 0.0842 | 13 | 0.0382 |
| 14 | 0.1126 | 14 | 0.0651 | 14 | 0.0710 | 14 | 0.0365 |
| 15 | 0.1000 | 15 | 0.0619 | 15 | 0.1152 | 15 | 0.0392 |
| 16 | 0.1196 | 16 | 0.0726 | 16 | 0.0549 | 16 | 0.0568 |
| 17 | 0.1228 | 17 | 0.0653 | 17 | 0.0457 | 17 | 0.0331 |
| 18 | 0.1111 | 18 | 0.0542 | 18 | 0.0418 | 18 | 0.0547 |
| 19 | 0.1096 | 19 | 0.0419 | 19 | 0.0482 | 19 | 0.0459 |
| 20 | 0.1032 | 20 | 0.0693 | 20 | 0.0658 | 20 | 0.0447 |
| 21 | 0.0982 | 21 | 0.0637 | 21 | 0.0548 | 21 | 0.0452 |
| 22 | 0.1163 | 22 | 0.0866 | 22 | 0.0550 | 22 | 0.0260 |
| 23 | 0.1051 | 23 | 0.0550 | 23 | 0.0524 | 23 | 0.0208 |
| 24 | 0.1141 | 24 | 0.0338 | 24 | 0.0586 | 24 | 0.0426 |
| 26 | 0.0969 | 25 | 0.0167 | 25 | 0.0517 | 25 | 0.0283 |
| 27 | 0.1079 | 26 | 0.0348 | 26 | 0.0869 | 26 | 0.0292 |
| 29 | 0.0813 | 27 | 0.0214 | 27 | 0.0660 | 27 | 0.0527 |
| 30 | 0.0690 | 28 | 0.0582 | 28 | 0.0691 | 28 | 0.0278 |
| 31 | 0.1274 | 29 | 0.0575 | 29 | 0.0549 | 29 | 0.0207 |
| 32 | 0.0659 | 30 | 0.0511 | 30 | 0.0739 | 30 | 0.0069 |
| 33 | 0.0852 | 31 | 0.0466 | 31 | 0.0276 | 31 | 0.0258 |
| 34 | 0.0713 | 32 | 0.0131 | 32 | 0.0685 | 32 | 0.0378 |
| 36 | 0.1004 | 33 | 0.0322 | 33 | 0.0436 | 33 | 0.0338 |
| 38 | 0.0730 | 34 | 0.0152 | 34 | 0.0432 | 34 | 0.0178 |
| 39 | 0.1174 | 36 | 0.0619 | 35 | 0.0471 | 35 | 0.0303 |
| 40 | 0.0835 | 37 | 0.0521 | 37 | 0.0465 | 37 | 0.0325 |
| 41 | 0.0368 | 38 | 0.0142 | 38 | 0.0832 | 39 | 0.0418 |
| 45 | 0.0614 | 40 | 0.0288 | 39 | 0.0304 | 40 | 0.0228 |
| 46 | 0.0556 | 41 | 0.0268 | 42 | 0.0656 | 41 | 0.0274 |
| 47 | 0.0740 | 43 | 0.0388 | 44 | 0.0196 | 42 | 0.0192 |
| 49 | 0.0604 | 45 | 0.0551 | 45 | 0.0414 | 43 | 0.0437 |
| 50 | 0.0359 | 47 | 0.0472 | 48 | 0.0328 | 45 | 0.0265 |
| 51 | 0.0649 | 49 | 0.0238 | 51 | 0.0235 | 46 | 0.0348 |
| 56 | 0.0539 | 50 | 0.0494 | 52 | 0.0494 | 48 | 0.0093 |
| 60 | 0.0518 | 51 | 0.0224 | 56 | 0.0425 | 49 | 0.0026 |
| 61 | 0.0246 | 52 | 0.0434 | 57 | 0.0536 | 50 | 0.0269 |
| 62 | 0.0643 | 54 | 0.0238 | 58 | 0.0422 | 59 | 0.0111 |
| 74 | 0.0639 | 55 | 0.0389 | 60 | 0.0424 | 64 | 0.0360 |
| 78 | 0.0361 | 57 | 0.0244 | 63 | 0.0584 | 77 | 0.0229 |
| 81 | 0.0413 | 64 | 0.0310 | 67 | 0.0570 | 78 | 0.0208 |
| 93 | 0.0182 | 65 | 0.0507 | 68 | 0.0252 | 81 | 0.0231 |
| 99 | 0.0256 | 76 | 0.0156 | 73 | 0.0330 | 82 | 0.0244 |
| 125 | 0.0337 | 82 | 0.0343 | 76 | 0.0387 | 99 | 0.0090 |
| 144 | 0.0277 | 88 | 0.0152 | 78 | 0.0240 | 135 | 0.0077 |
| 153 | 0.0237 | 108 | 0.0109 | 82 | 0.0336 | 148 | 0.0048 |
| 156 | 0.0250 | 122 | 0.0173 | 93 | 0.0359 | 230 | 0.0045 |
| 184 | 0.0077 | 125 | 0.0085 | 98 | 0.0311 | 506 | 0.0027 |
| 188 | 0.0170 | 130 | 0.0070 | 99 | 0.0334 | - | - |
| 201 | 0.0121 | 140 | 0.0157 | 103 | 0.0176 |  |  |
| 204 | 0.0191 | 159 | 0.0102 | 111 | 0.0257 |  |  |
| 249 | 0.0118 | 166 | 0.0080 | 117 | 0.0251 |  |  |
| 381 | 0.0091 | 184 | 0.0105 | 121 | 0.0247 |  |  |
| - | - | 233 | 0.0133 | 127 | 0.0167 |  |  |
|  |  | 234 | 0.0083 | 128 | 0.0273 |  |  |
|  |  | 235 | 0.0078 | 131 | 0.0112 |  |  |
|  |  | 497 | 0.0030 | 134 | 0.0287 |  |  |
|  |  | - | - | 148 | 0.0211 |  |  |
|  |  |  |  | 158 | 0.0201 |  |  |
|  |  |  |  | 243 | 0.0105 |  |  |

1. **Chunk length distributions**

| Time Period 1 | | Time Period 2 | | Time Period 3 | | Time Period 4 | |
| --- | --- | --- | --- | --- | --- | --- | --- |
| Chunk length | Tokens | Chunk length | Tokens | Chunk length | Tokens | Chunk length | Tokens |
| 1 | 260 | 1 | 128 | 1 | 129 | 1 | 177 |
| 2 | 252 | 2 | 327 | 2 | 215 | 2 | 272 |
| 3 | 460 | 3 | 725 | 3 | 331 | 3 | 221 |
| 4 | 608 | 4 | 739 | 4 | 400 | 4 | 202 |
| 5 | 357 | 5 | 355 | 5 | 343 | 5 | 172 |
| 6 | 251 | 6 | 211 | 6 | 265 | 6 | 144 |
| 7 | 144 | 7 | 77 | 7 | 176 | 7 | 145 |
| 8 | 71 | 8 | 28 | 8 | 114 | 8 | 110 |
| 9 | 28 | 9 | 20 | 9 | 58 | 9 | 104 |
| 10 | 19 | 10 | 7 | 10 | 29 | 10 | 51 |
| 11 | 5 | 11 | 3 | 11 | 21 | 11 | 61 |
| 12 | 5 | - | - | 12 | 16 | 12 | 34 |
| - | - |  |  | 13 | 7 | 13 | 41 |
|  |  |  |  | 14 | 3 | 14 | 18 |
|  |  |  |  | 15 | 1 | 15 | 21 |
|  |  |  |  | 16 | 1 | 16 | 8 |
|  |  |  |  | - | - | 17 | 3 |
|  |  |  |  |  |  | 18 | 3 |
|  |  |  |  |  |  | 19 | 1 |
|  |  |  |  |  |  | 20 | 4 |
|  |  |  |  |  |  | 21 | 4 |
|  |  |  |  |  |  | 23 | 2 |
|  |  |  |  |  |  | 25 | 2 |
|  |  |  |  |  |  | 26 | 2 |

1. **Top 10 hub words in each network**

| Network 1 | | Network 2 | |
| --- | --- | --- | --- |
| Word | Network Centrality | Word | Network Centrality |
| “之” | 0.1780 | “之” | 0.1415 |
| “而” | 0.1094 | “不” | 0.0666 |
| “者” | 0.0771 | “为” | 0.0586 |
| “不” | 0.0752 | “有” | 0.0482 |
| “其” | 0.0642 | “其” | 0.0391 |
| “於” | 0.0619 | “而” | 0.0374 |
| “有” | 0.0536 | “以” | 0.0352 |
| “以” | 0.0471 | “者” | 0.0344 |
| “也” | 0.0444 | “所” | 0.0243 |
| “为” | 0.0428 | “于” | 0.0212 |
| Network 3 | | Network 4 | |
| Word | Network Centrality | Word | Network Centrality |
| “了” | 0.0867 | “的” | 0.2987 |
| “是” | 0.0581 | “和” | 0.0947 |
| “不” | 0.0567 | “在” | 0.0599 |
| “一” | 0.0477 | “了” | 0.0566 |
| “得” | 0.0445 | “中国” | 0.0442 |
| “人” | 0.0441 | “要” | 0.0369 |
| “的” | 0.0438 | “是” | 0.0354 |
| “他” | 0.0419 | “为” | 0.0269 |
| “去” | 0.0362 | “健康” | 0.0267 |
| “来” | 0.0330 | “发展” | 0.0239 |

1. * Corresponding author: lhtzju@yeah.net [↑](#footnote-ref-1)
